# Portuguese Psychologists' Attitudes Toward Internet Interventions: Exploratory Cross-Sectional Study

**DOI:** 10.2196/16817

**Published:** 2020-04-06

**Authors:** Cristina Mendes-Santos, Elisabete Weiderpass, Rui Santana, Gerhard Andersson

**Affiliations:** 1 Department of Culture and Society Linköping University Linköping Sweden; 2 NOVA National School of Public Health Public Health Research Centre Universidade Nova de Lisboa Lisboa Portugal; 3 International Agency for Research on Cancer Lyon France; 4 Department of Behavioural Sciences and Learning Linköping University Linköping Sweden; 5 Department of Clinical Neuroscience Psychiatry Section Karolinska Institutet Stockholm Sweden

**Keywords:** attitudes, psychologists, e-mental health, internet interventions, Attitudes Toward Internet Interventions Survey (ATIIS), Portugal, EU

## Abstract

**Background:**

Despite the significant body of evidence on the efficacy and cost-effectiveness of internet interventions, the implementation of such programs in Portugal is virtually non-existent. In addition, Portuguese psychologists’ use and their attitudes towards such interventions is largely unknown.

**Objective:**

The aim of this study was to explore Portuguese psychologists’ knowledge, training, use and attitudes towards internet interventions; to investigate perceived advantages and limitations of such interventions; identify potential drivers and barriers impacting implementation; and study potential factors associated to previous use and attitudes towards internet interventions.

**Methods:**

An online cross-sectional survey was developed by the authors and disseminated by the Portuguese Psychologists Association to its members.

**Results:**

A total of 1077 members of the Portuguese Psychologists Association responded to the questionnaire between November 2018 and February 2019. Of these, 37.2% (N=363) were familiar with internet interventions and 19.2% (N=188) considered having the necessary training to work within the field. 29.6% (N=319) of participants reported to have used some form of digital technology to deliver care in the past. Telephone (23.8%; N=256), e-mail (16.2%; N=175) and SMS (16.1%; N=173) services were among the most adopted forms of digital technology, while guided (1.3%; N=14) and unguided (1.5%; N=16) internet interventions were rarely used. Accessibility (79.9%; N=860), convenience (45.7%; N=492) and cost-effectiveness (45.5%; N=490) were considered the most important advantages of internet interventions. Conversely, ethical concerns (40.7%; N=438), client’s ICT illiteracy (43.2%; N=465) and negative attitudes towards internet interventions (37%; N=398) were identified as the main limitations. An assessment of participants attitudes towards internet interventions revealed a slightly negative/neutral stance (Median=46.21; SD=15.06) and revealed greater acceptability towards blended treatment interventions (62.9%; N=615) when compared to standalone internet interventions (18.6%; N=181). Significant associations were found between knowledge (χ^2^_4_=90.4; *P*<.001), training (χ^2^_4_=94.6; *P*<.001), attitudes (χ^2^_3_=38.4; *P*<.001) and previous use of internet interventions and between knowledge (χ^2^_12_=109.7; *P*<.001), training (χ^2^_12_=64.7; *P*<.001) and attitudes towards such interventions, with psychologists reporting to be ignorant and not having adequate training in the field, being more likely to present more negative attitudes towards these interventions and not having prior experience in its implementation.

**Conclusions:**

This study revealed that most Portuguese psychologists are not familiar with and have no training or prior experience using internet interventions and had a slightly negative/neutral attitude towards such interventions. There was greater acceptability towards blended treatment interventions compared to standalone internet interventions. Lack of knowledge and training were identified as the main barriers to overcome, underlining the need of promoting awareness and training initiatives to ensure internet interventions successful implementation.

## Introduction

### Background

Advances in digital technology are transforming the current health care delivery paradigm, enabling health systems to overcome physical and organizational barriers and creating an opportunity to deliver accessible and convenient mental health care services at a distance [1]. In recent years, the development of internet interventions—self-help guided or unguided interventions based on established psychotherapy models operated via secure platforms or mobile apps that aim at providing synchronous or asynchronous health and mental health–related assistance—has generated significant evidence of efficacy and cost effectiveness [2-9]. There are several advantages associated with implementing internet interventions [10-15]: (1) low-threshold accessibility and dissemination potential, (2) high use flexibility and adaptability, (3) standardized structure and integrated treatment monitoring, (4) self-efficacy and patient empowerment promotion, (5) high level of anonymity and privacy, and (6) low delivery costs.

Despite evidence of efficacy and potential usefulness, implementation of internet interventions in clinical practice has been peculiarly slow [16]. In Portugal, a country characterized by a significant mental health treatment gap where treatment median delay reaches 23 years for anxiety disorders [17], internet interventions could represent an effective opportunity to reach those in need. Yet such interventions are virtually nonexistent. This fact may be related to potential barriers and limitations of internet interventions such as the absence of an adequate legal and regulatory framework for providing mental health care via the internet, health care professional and patient information and communication technologies (ICT) illiteracy, possible technological problems, potential security breaches associated with computerized systems, and health care provider and patient attitudes toward internet interventions [13]. As potential end users and prescribers of a variety of e-mental health programs (eg, guided and unguided internet interventions, apps, serious games), psychologists might play a crucial role in the uptake and dissemination of internet interventions. Thus, understanding attitudes toward such programs is key to identify drivers and barriers to implementation and overcome limitations to dissemination.

### Prior Work

Previous studies have been performed to investigate psychologist attitudes toward internet interventions and categorize drivers and barriers to the adoption of such programs. Overall, findings suggest that therapist attitudes range from neutral and cautiously positive to generally positive [18], and several factors impact adoption.

In a study performed by Mora and colleagues [19] querying members of the New York State Psychological Association, theoretical orientation was found to predict the use of internet-based interventions. Dynamic and existential oriented therapists were less likely to endorse internet interventions and more likely to have negative attitudes toward them than therapists from other theoretical stances such as cognitive behavioral, cognitive, behavioral, and systems therapists, a finding that has been corroborated in other studies [20-24]. Another study performed by Simms and colleagues [25], focusing on Canadian mental health professional perceptions of telemental health, found overall positive attitudes particularly for clients in remote and rural locations and people with disabilities, and factors predicting frequency of use of telemental health were having performed previous training in the field, working within mental health for longer, and considering technology as easy to use. Corroborating these findings, a study by Bruno et al [26] focusing on Australian health professional attitudes reported that professionals with higher perceptions of usefulness and ease of use of internet-supported psychological interventions presented more positive attitudes toward using such interventions than professionals with lower perceptions of usefulness and ease of use. Moreover, the possibility of encouraging clients to develop self-management skills and reaching clients who might not otherwise engage in therapy were considered the main benefits of internet-supported psychological interventions by participants in this study. More recently, Feijt et al [15], in a qualitative study involving Dutch psychologists, found them to believe e-mental health brings new treatment possibilities (eg, virtual reality and biofeedback) and may accelerate the treatment process (mediated contact in-between regular sessions may intensify treatment and allows for the introduction of new therapeutic elements earlier in the process), reinforcing intimacy in the therapeutic relationship.

Contrasting with these findings, a more guarded attitude was identified in other studies, and major concerns related to security and ethical and legal requirements have been reported as important barriers to adoption [15,21,27-29]. Lack of clarity and knowledge regarding ethical and regulatory requirements emerged as important limitations in studies by Perle et al [21] and Glueckauf et al [27], and a possible threat to confidentiality and therapeutic boundaries posed by online communication was identified by Evans et al [28]. A further concern expressed in this study was that the therapeutic alliance could be negatively affected due to missing nonverbal cues in communication. This was also a finding in other studies [19,29].

Technical barriers such as connection challenges and disruptions were also flagged as potential limitations of internet interventions, and a major concern reported in several studies [19,21,27] relates to handling emergencies and managing crisis situations (eg, suicide ideation, child abuse) in the context of online practice. In a study by Glueckauf et al [27], over half of respondents reported inadequate skills in managing crisis situations in this environment. In addition, Perle and colleagues [21] reported that adoption of telehealth depended on disorder type and was rejected for disorders considered difficult to treat even face-to-face, such as schizophrenia. Likewise, Vigerland et al [22] found Swedish mental health professionals to be strong supporters of computerized cognitive behavior therapy for preventing and treating mild to moderate problems, but more caution was reported regarding severe mental health problems.

Other important findings reported in previous research as influencing therapist attitudes relate to demographic and background factors (eg, age, gender, length of professional career, personal experience in using modern technologies) [19,21,30], practical concerns (eg, costs of setting up and maintaining the necessary infrastructure) [15], fear of replacement [31,32], social influence [33], skepticism about feasibility of delivery within existing care services [32,34], forces within the care system, design and usability [15], low patient engagement, and difficulties in managing comorbidities [32].

In Portugal, only one study addressed psychologist attitudes toward electronic psychological interventions (EPI). In the study, Neves et al [24] showed that Portuguese psychologists reported moderately less favorable attitudes toward EPI. Moreover, years of termination of vocational training and cognitive behavioral and eclectic/integrative theoretical orientations were predictors of positive attitudes toward EPI. Nevertheless, a full report on the findings of this study is unavailable for consultation, and gaps persist in our knowledge regarding Portuguese psychologists’ use and attitudes toward internet interventions, perceived advantages and limitations of such interventions, drivers and barriers impacting implementation, and factors associated to adoption of internet interventions in the country.

### Aim

In spite of the findings that psychologists’ attitudes toward internet interventions appear to be positive, there are variations between countries [34] and lack of consensus between studies regarding factors influencing adoption. The aim of this study was to explore Portuguese psychologists’ knowledge, training, use and attitudes toward internet interventions; to investigate perceived advantages and limitations of such interventions; identify potential drivers and barriers impacting implementation; and study potential factors associated to previous use and attitudes toward internet interventions.

## Methods

### Study Design and Procedures

This study was conducted in the framework of the iNNOVBC (A Guided Internet-Delivered Individually Tailored ACT-Influenced Cognitive Behavioral Intervention to Improve Psychosocial Outcomes in Breast Cancer Survivors) project (ClinicalTrials.gov NCT03275727) [35] and approved by the Portuguese Data Protection Committee (approval number: 10727/2017) and Portuguese Psychologists Association (Ordem dos Psicólogos Portugueses, or OPP) ethical committee, adopting an exploratory cross-sectional design. An anonymous online self-report questionnaire located on the Web-based survey platform LimeSurvey [36] was disseminated by OPP via email to its members. As being registered with OPP is a requirement to practice psychology in Portugal and email is the institutional channel of communication with its members, the whole universe of licensed psychologists was reached using this method. Participants initially accessed an introduction to the study and informed consent form, followed by a link providing access to the questionnaire. No follow-up reminders were sent to recipients of the questionnaire. The time frame of data collection was November 2018 to February 2019. After conclusion of the recruitment period, researchers exported data from LimeSurvey [36] to SPSS Statistics version 24.0 (IBM Corp).

### Survey Development and Design

Due to the scarcity of adequate instruments designed to evaluate the outlined issues in the target population, the Attitudes Toward Internet Interventions Survey (ATIIS) was developed. After a comprehensive literature review was performed, most relevant publications were identified and served as a basis for development [20,21,26,34,37-40]. A preliminary version of this self-report questionnaire was created, pilot-tested with 3 participants, and subsequently checked by researchers and clinicians with some experience within the field of internet interventions and OPP. Changes were made in line with suggestions emerging from this process, and final item selection was completed by the authors. Selection criteria were redundancy, relevance of items, and face validity.

The final version of the survey comprised 38 items assessing 4 main categories: (1) information relating to frequency of use of digital technology and internet interventions in practice (eg, use and frequency of use of digital technology, provision and prescription of internet interventions, contexts and purposes of use); (2) knowledge and training within the internet interventions field; (3) perceived advantages and limitations of internet interventions and potential barriers and challenges impacting implementation; and (4) attitudes toward internet interventions (eg, related to efficacy and efficiency; privacy, security and confidentiality; patient empowerment and increased disinhibition; therapeutic processes and alliance; and blended, complementary, and stand-alone interventions). The attitudes section was composed of 21 items aimed at capturing cognitive, affective, and behavioral predispositions of favor or disfavor [41] toward internet interventions.

Demographic and background items were added to the questionnaire to gather supplementary information (eg, age, gender, educational and professional background, professional experience, and theoretical orientation). The survey questions were asked in the form of dichotomous and multiple choice questions and in the form of 5-point (0=completely disagree to 5=completely agree) Likert scales. Since it was not expected for participants to be familiar with the concept of internet interventions, an explanation of the concept based on the definition by Barak et al [42] was provided in the instructions section of the questionnaire.

An assessment of the validity of the attitudes section of the questionnaire resulted in 21 items clustering in two dimensions labeled as positive attitudes and negative attitudes. Reliability of the scale was also tested and considered excellent (α=.91). A detailed description of the psychometric properties assessment process of this scale and its results can be found below. A copy of the instrument is available in Multimedia Appendix 1.

### Statistical Analysis

Statistical analyses were divided into 4 steps and conducted using SPSS Statistics. First, descriptive statistics such as frequency distributions, measures of variability, and measures of central tendency were calculated to characterize the study sample and determine its face validity. These statistics encompassed demographic and background characteristics such as, age, gender, educational and professional background, professional experience (in years), and theoretical orientation.

Second, a psychometric properties evaluation process of ATIIS took place, and an exploratory factor analysis (EFA) based on the principal component analysis method using a varimax rotation was conducted to determine the factor structure of the questionnaire, perform scale purification, and determine the questionnaire’s construct validity. The whole study sample (1077) was used for this purpose. The Kaiser-Meyer-Olkin (KMO) test and a Bartlett test of sphericity were calculated to measure sampling adequacy (confirmed if KMO value greater than .5) and appropriateness of the extracted factors (significant at *P*<.05), respectively. The initial model hypothesized that items would load on either a positive or a negative factor, and items with factor loadings above .40 were considered acceptable [43]. Scores on the negative items were reversed, and dimension scores were weighted, summed, and rescaled on a 100-point scale to simplify interpretation and obtain a continuous indicator of attitude toward internet interventions. Higher scores indicated a more positive attitude. The final version of ATIIS was then subject to a reliability analysis based on the computation of internal consistency (Cronbach alpha).

Following this process, results pertaining to frequency of use of digital technology and internet interventions in daily practice, provision and prescription of internet interventions, contexts and purposes of use, perceived advantages and limitations of internet interventions, and potential barriers and challenges impacting implementation were analyzed. Although some of the questionnaire items contained multiple response options for which up to 3 response categories could be selected, only single response options (eg, percentage of psychologists using chat services) rather than combined response options (eg, percentage of psychologists using chat services and videoconference) were calculated in order to simplify the analysis.

Finally, psychologist attitudes toward internet interventions were examined using descriptive statistics, and chi-square analysis and post hoc tests were used to determine if demographic (eg, sex and age) and background factors (eg, academic background, work context, years of professional experience, theoretical orientation), knowledge, training, previous experience of use, recommendation, future use, and attitudes toward internet interventions would be associated and differed between participants holding extreme attitudes toward internet interventions.

## Results

### Participants and Recruitment

The total sample comprised 1077 members of the OPP recruited between November 2018 and February 2019. Considering the number of psychologists registered as members at the time (21,214, data provided by T Pereira, OPP’s head of cabinet), response rate was 5.08%. Although we cannot determine the representativeness of the sample (OPP’s members demographic and background information is not available for consultation), demographic characteristics are similar to those published in the last census performed by OPP [44]. In this census, the mean age of Portuguese psychologists was 38 years, and 84.2% were female. On average, psychologists had 11 years of professional experience, and the majority held a license and/or master’s degree. Only 7% held a doctoral degree.

In our study sample, 91.6% (987/1077) of respondents were female, and age ranged from 20 to 77 years (mean 38.21; SD 9.49 years). Most participants held a license and/or master’s degree (722/1077, 67.0%), followed by postgraduate (273/1077, 25.3%), doctoral (75/1077, 7%), and bachelor’s degrees (7/1077, 0.6%). The majority of participants were active (986/1077, 91.6%) and worked primarily in private practice (270/1077, 25.1%), educational/research institutions (252/1077, 23.4%), and charities/nonprofit organizations (208/1077, 19.3%). Only 6.3% (68/1077), 4.4% (47/1077), and 1.6% (17/1077) of psychologists worked in the National Health Service (NHS) at primary, secondary and tertiary care, respectively. As for the length of time working within the field of psychology, the sample was evenly distributed, with 12.6% (136/1077) of professionals working for less than a year; 21.0% (226/1077) practicing psychology from 2 to 5 years, 17.3% (186/1077) working between 6 to 10 years in the field, 20.0% (215/1077) practicing between 11 to 15 years, 15.4% (166/1077) working from 16 to 20 years in this domain, and 13.7% (148/1077) practicing psychology for more than 21 years. Cognitive behavioral therapy was the most common theoretical orientation (56.0%, 603/1077), with psychodynamic (14.8%, 159/1077) and eclectic (13.5%, 145/1077) orientations being second and third.

### Attitudes Toward Internet Interventions Survey Psychometric Properties Assessment

In order to test the psychometric properties of the attitudes section of ATIIS, we explored its construct validity and reliability.

#### Construct Validity

An EFA based on principal component analysis and using a varimax rotation was conducted with the purpose of finding the underlying latent factors of ATIIS and determining the questionnaire’s construct validity. The whole study sample (N=1077) was used in this analysis. A KMO=.93 confirmed the sampling adequacy, and a Bartlett test of sphericity, χ^2^_210_=8003.39 (*P*<.001), indicated a possible statistically significant interrelationship between variables and, therefore, confirmed the factorial analysis validity to perform factor reduction.

The initial EFA resulted in 4 factors with eigenvalues above a Kaiser criterion of 1. However, a scree plot analysis revealed inflexions compatible with the retention of two factors. Due to convergence with theory, two factors were retained for the final EFA. The initial model hypothesized that items would load on either a positive or a negative factor. Total variance explained by these two factors was 44.10% (unrotated solution: factor one 36.2% and factor two 7.97% or rotated solution: factor one 22.40% and factor two 21.73%), and items clustering on these two factors suggested that the questionnaire measures two dimensions, labeled as positive attitudes (range of factor loadings: .375-.712) and negative attitudes (range of factor loadings: .459-.708). Items with factor loadings above *r*=.4 were considered as acceptable [43]. Scores on the negative items were reversed, and dimension scores were weighted, summed, and rescaled on a 100-point scale to simplify interpretation and obtain a continuous indicator of attitude toward internet interventions. Higher scores indicated a more positive attitude. A copy of the final questionnaire and item loading factors is presented in Multimedia Appendix 1.

#### Reliability

ATIIS reliability was assessed via the computation of Cronbach alpha. ATIIS total scale revealed excellent (α=.91) internal consistency and its subscales, positive (.88) and negative (.82) attitudes, showed good internal consistency [45].

### Portuguese Psychologists Reported Knowledge About Internet Interventions

An examination of collected data indicated that 37.2% (363/978) of respondents were familiar with the concept of providing psychological support via the internet. Nevertheless, a narrower group reported knowing how these types of interventions work (218/978, 22.3%), and only 19.2% (188/978) were considered to have the necessary training to work in the field.

### Frequency of Use of Digital Technology and Internet Interventions in Daily Practice

Around 29.6% (319/1077) of participants reported that they use or have used in the past some form of digital technology to provide support in the context of their practice. Of nonusers (758/1077, 70.4%), 61.7% (468/758) reported to be considering using it in the future. Telephone (256/1077, 23.8%), email (175/1077, 16.2%,) and short message service (SMS) or text message (173/1077, 16.1%) services were among the most used forms of digital technology, while chat services (66/1077, 6.1%) and unguided (16/1077, 1.5%) and guided (14/1077, 1.3%) internet interventions were much less used. However, 8.7% (94/1077) reported using videoconference services. Digital technology was mostly used by clinical and health psychologists (269/319, 84.3%), followed by educational psychologists (31/319, 9.7%). In most cases, digital technology was used as a complement to face-to-face interventions (288/319, 90.3%) rather than as a stand-alone interventions (31/319, 9.7%) for the purpose of treating mental health disorders such as anxiety or depression (205/319, 64.3%). Increasing accessibility to information and psychological care was reported as the main reason for using digital technology in practice by 54.5% (174/319) of respondents whereas only 0.6% (2/319) used it for research (see Table 1).

**Table 1:** Motivations for previous use of digital technology in psychological practice (n=319).

Motivation	Value, n (%)
Increasing accessibility to information and psychological care	174 (54.5)
Lowering the costs of psychological interventions	3 (0.9)
Increasing adherence to psychological interventions	61 (19.1)
Monitoring treatment progress	41 (12.9)
Facilitating follow-up care	26 (8.2)
Managing crisis situations	5 (1.6)
Improving career prospects	5 (1.6)
Research	2 (0.6)
Other	2 (0.6)

Almost a fifth (19.1%, 206/1077) reported that they recommend or have recommended in the past to their clients accessing online services or resources with the aim of improving their emotional wellbeing and/or health status. Most frequently recommended resources were websites providing information about mental and/or somatic health (57.3%, 118/206), blogs, discussion forums and social networks (38.3%, 79/206), videoconference-delivered psychological interventions (29.1%, 60/206), and apps (28.2%, 58/206). On the other hand, online support groups (21.8, 45/206) and guided (13.1%, 27/206) and unguided (4.4%, 9/206) internet interventions were the least recommended. Only a minority of respondents (3.7%, 39/1077) provided or recommended internet interventions to their clients in a regular basis.

### Advantages and Limitations Associated With Internet Interventions

Considering the potential advantages of internet interventions, accessibility (860/1077, 79.9%), convenience (492/1077, 45.7%), and cost effectiveness (490/1077, 45.5%) of such interventions were considered the most important advantages. Conversely, ethical concerns (438/1077, 40.7%), client ICT illiteracy (465/1077, 43.2%), and client negative attitudes toward internet interventions (398/1077, 37.0%) were identified as the main limitations. Other advantages and limitations associated with internet interventions are presented in Table 2.

**Table 2:** Advantages and limitations associated with internet interventions (n=1077).

Characteristic		Value, n (%)
**Advantage**		
	Accessibility	860 (79.9)
	Convenience	492 (45.7)
	Economical (cost effectiveness and sustainability to health care systems)	490 (45.5)
	Reduced stigma associated with psychological support/confidentiality	206 (19.1)
	Privacy/anonymity	164 (15.2)
	Health equity	142 (13.2)
	Client empowerment	131 (12.2)
	Personalized health care	89 (8.3)
	None	77 (7.1)
	Scientific evidence	43 (4)
**Limitations**		
	Client information and communications technologies illiteracy	465 (43.2)
	Ethical	438 (40.7)
	Client attitudes toward internet interventions	398 (37.0)
	Information systems security	386 (35.8)
	Cultural	271 (25.5)
	Therapist attitudes toward internet interventions	259 (24.0)
	Health care systems not ready for implementation	234 (21.7)
	Cost and accessibility to digital technology	144 (13.4)
	Therapist information and communications technologies illiteracy	96 (8.9)
	Other	82 (7.5)
	Political (decision makers not interested in implementation)	50 (4.6)
	Economical (cost effectiveness and sustainability to health care systems)	18 (1.7)
	None	18 (1.7)

When questioned about the possibility of internet interventions presenting more disadvantages than advantages, only 24.5% (239/1077) of participants refuted this claim.

### Barriers to Implementation of Internet Interventions

The main barriers to overcome in the implementation of internet interventions were related to limitations on the conceptual comprehension and implementation of self-help techniques by clients (676/1077, 62.8%), therapist perceptions of insufficient scientific evidence on the efficacy and cost effectiveness of internet interventions (670/1077, 62.2%), limitations on the adaptation of treatment protocols (665/1077, 61.7%), patient ICT illiteracy (516/1077, 47.9%), and low adherence both from patients (466/1077, 43.3%) and psychologists (437/1077, 40.6%) toward such programs. Negative attitudes presented both by clients (417/1077, 38.7%) and therapists (416/1077, 38.6%) were also considered an important obstacle to overcome in the implementation of internet interventions (see Table 3).

**Table 3:** Barriers to implementation of internet interventions (n=1077).

Characteristic		Value, n (%)
**Faced by clients**		
	Ability to comprehend concepts and learn self-help techniques	676 (62.8)
	Client information and communications technologies illiteracy	516 (47.9)
	Low adherence	466 (43.3)
	Negative attitudes	417 (38.7)
	Scientific evidence (efficacy and cost effectiveness)	362 (33.6)
	Costs and access to digital technology and information technology infrastructures	211 (19.6)
	Time consumption	28 (2.6)
	None	21 (1.9)
**Faced by therapists**		
	Scientific evidence (efficacy and cost effectiveness)	670 (66.2)
	Adaptation of treatment protocols to the digital environment	665 (61.7)
	Low adherence	437 (40.6)
	Negative attitudes	416 (38.6)
	Clinician information and communications technologies illiteracy	178 (16.5)
	Costs and access to digital technology and information technology infrastructures	91 (8.4)
	Time consumption	38 (3.5)
	None	20 (1.9)

### Analysis of Portuguese Psychologist Attitudes Toward Internet Interventions

The median score on the ATIIS scale was 46.21 (SD 15.06), which corresponds to a slightly negative/neutral attitude toward internet interventions. Factors contributing to this predisposition relate to possible security (417/1077, 42.6%) and confidentiality (494/1077, 50.5%) breaches when using internet interventions, reported discomfort about dealing with sensitive information online (466/1077, 47.7%), perceived inaccuracy of remote psychological assessment processes (578/1077, 59.1%), perceived unsuitability of internet interventions for crisis management (473/1077, 48.4%), a disbelief on the possibility of establishing therapeutic alliance via the internet (356/1077, 36.5%), and a generalized perception of face-to-face interventions as being superior for client education/self-management skills development (702/1077, 71.8%) and mental disorders treatment (699/1077, 71.6%) compared with internet interventions. Absence of knowledge about the efficacy (561/1077, 57.4%) and efficiency (443/1077, 45.3%) of internet interventions and its impact on patient empowerment (461/1077, 47.2%) and a possible loss of control of the therapeutic process by clinicians (372/1077, 38.1%) also seem to influence this stance. Nevertheless, perceived convenience (473/1077, 45.6%) of internet interventions, encouragement of emotional expression in some cases (360/1077, 36.8%), facilitation of the follow-up process (440/1077, 45%), and the possibility of delivering blended (615/978, 62.9%) and pharmacotherapy complementary interventions (511/978, 52.2%) rather than stand-alone internet interventions (181/978, 18.6%) seem to balance attitudes regarding this matter.

### Factors Associated With Previous Experience of Use and Attitudes Toward Internet Interventions

Chi-square tests and post hoc analyses were performed to examine possible associations between demographic factors (sex, age), background factors (academic background, work context, years of professional experience, theoretical orientation), knowledge, training, recommendation, future use, previous experience of use, and attitudes toward internet interventions. Differences in responses between participants with or without prior experience of use and holding extreme attitudes toward these interventions were also evaluated. Percentiles (assumed here as the percentage of scores that fall below the scores of interest) were computed to categorize participant attitudes and identify those who held extremely negative (scores <Q1=36.03, 244; 25.1%) and extremely positive (scores >Q3=53.49, 242; 24.9%) attitudes toward internet interventions.

Chi-square analyses (see Table 4) revealed a significant association between previous experience of use and age, theoretical orientation, work context, years of professional experience, knowledge, training, recommendation, and attitudes toward internet interventions.

Considering age, psychologists aged between 41 and 60 years were more likely to have used the telephone or internet to provide psychological support in the past, while psychologists aged 30 years and younger were less likely to have done it. Similarly, psychologists with less than 5 years of professional experience were less likely to have already used such interventions, whereas psychologists with more than 16 years of professional experience were more likely to have used internet interventions in the past. Work context also seems to impact the use of internet interventions. Participants working at the NHS and in private practice had a higher probability of using the internet and telephone to provide care. Working at public services, education/research facilities, and charities made it less probable participants had adopted these interventions.

Regarding self-reported knowledge and training on internet interventions, psychologists reporting moderate to high knowledge and training were more likely to have prior experience in implementing such programs than those whom reported little to no knowledge about internet interventions. Furthermore, having a psychodynamic theoretical orientation impacted use positively, making it more likely that psychodynamic psychologists had used internet interventions in the past than expected. No significant associations were found between other theoretical stances and internet intervention adoption.

Extreme attitudes toward internet interventions seem, as well, to have significantly impacted adoption. Psychologists presenting more negative attitudes toward these interventions were less likely to have prior experience using internet interventions than expected and when compared with psychologists holding more positive attitudes. Finally, prior experience implementing internet interventions significantly affected referrals and the possibility of psychologists recommending such programs to their clients. Psychologists with prior experience of use were more likely to recommend internet interventions and online resources with the purpose of improving their clients’ health status.

**Table 4:** Factors associated to previous experience of use.

Characteristic		Previous experience of use^a^		Chi-square tests		
		No	Yes	*P* value	Chi-square	Cramér V Φc
**Age in years (n=1077)**				**<.001**	**χ^2^_4_=42.4**	**.20**
	≤30	207 (4.4)	47 (–4.4)	—	—	—
	31-40	323 (1.4)	121 (–1.4)	—	—	—
	41-50	152 (–3.5)	95 (3.5)	—	—	—
	51-60	59 (–4.2)	52 (4.2)	—	—	—
	≥61	17 (1.1)	4 (–1.1)	—	—	—
**Theoretical orientation (n=1077)**				**<.001**	**χ^2^_6_=27.7**	**.16**
	Cognitive behavior therapy	435 (1.4)	168 (–1.4)	—	—	—
	Psychodynamic	99 (–2.4)	60 (2.4)	—	—	—
	Humanist	25 (–1.6)	17 (1.6)	—	—	—
	Eclectic	93 (–1.8)	52 (1.8)	—	—	—
	Systemic	24 (1.5)	5 (–1.5)	—	—	—
	Other	37 (0.1)	15 (–0.1)	—	—	—
	None	45 (3.9)	2 (–3.9)	—	—	—
**Work context (n=1077)**				**<.001**	**χ^2^_7_=50.9**	**.22**
	National Health Service	82 (–2.2)	50 (2.2)	—	—	—
	Private practice	151 (–6.0)	119 (6.0)	—	—	—
	Public services	59 (2.4)	12 (–2.4)	—	—	—
	Private companies	22 (1.0)	6 (–1.0)	—	—	—
	Rehabilitation services/prisons	21 (0.9)	6 (–0.9)	—	—	—
	Education/research institutions	190 (2.0)	62 (–2.0)	—	—	—
	Charities	164 (3.0)	44 (–3.0)	—	—	—
	Other	69 (1.5)	20 (–1.5)	—	—	—
**Professional experience in years (n=1077)**				**<.001**	**χ^2^_7_=49.0**	**.21**
	≤1	122 (5.3)	14 (–5.3)	—	—	—
	2-5	172 (2.1)	54 (–2.1)	—	—	—
	6-10	129 (–0.3)	57 (0.3)	—	—	—
	11-15	151 (–0.1)	64 (0.1)	—	—	—
	16-20	96 (–3.9)	70 (3.9)	—	—	—
	≥21	88 (–3.1)	60 (3.1)	—	—	—
**Self-reported knowledge^a^ (n=978)**				**<.001**	**χ^2^_4_=90.4**	**.30**
	Completely disagree	145 (6.1)	15 (–6.1)	—	—	—
	Moderately disagree	200 (3.6)	52 (–3.6)	—	—	—
	Neither agree nor disagree	143 (0)	60 (0)	—	—	—
	Moderately agree	172 (–5.1)	119 (5.1)	—	—	—
	Completely agree	29 (–5.8)	43 (5.8)	—	—	—
**Self-reported training^b^ (n=978)**				**<.001**	**χ^2^_4_=94.6**	**.31**
	Completely disagree	301 (7.4)	54 (–7.4)	—	—	—
	Moderately disagree	188 (0.1)	78 (–0.1)	—	—	—
	Neither agree nor disagree	115 (–0.8)	54 (0.8)	—	—	—
	Moderately agree	65 (–6.2)	71 (6.2)	—	—	—
	Completely agree	20 (–5.2)	32 (5.2)	—	—	—
**Recommendation^c^ (n=1077)**				**<.001**	**χ^2^_4_=37.3**	**.19**
	No	649 (6.1)	222 (–6.1)	—	—	—
	Yes	109 (–6.1)	97 (6.1)	—	—	—
**Attitudes (n=972)**				**<.001**	**χ^2^_3_=38.4**	**.20**
	≤36.02	190 (2.9)	54 (–2.9)	—	—	—
	36.03-46.20	185 (2.0)	60 (–2.0)	—	—	—
	46.21-53.48	177 (1.2)	64 (–1.2)	—	—	—
	≥53.49	133 (–6.1)	109 (6.1)	—	—	—

^a^Adjusted standardized residual frequencies appear in parentheses after observed group frequencies. Original wording: I am familiar with the concept of providing psychological support via the internet. Rated on a 5-point scale: 1=completely disagree to 5=completely agree.

^b^Original wording: I believe to have the necessary training to provide psychological support via the internet. Rated on a 5-point scale: 1=completely disagree to 5=completely agree.

^c^Original wording: Have you ever recommended the use of internet-based psychological support or other online resources in order to improve a client’s health status?

The association of attitudes toward internet interventions with demographic and background factors, knowledge, training, recommendation, and future use was also assessed via chi-square analyses (see Table 5). These tests revealed a significant association between attitudes of respondents and self-reported knowledge, self-reported training, previous experience of use, recommendation, and future use of internet interventions. No significant associations were found between attitudes of respondents and demographic or background factors such as age, theoretical orientation, or professional experience.

Findings in these analyses primarily reflect the fact that psychologists without any knowledge, training, or previous experience using internet interventions are more likely to present more negative attitudes toward these interventions than expected. Conversely, psychologists reporting moderate to high knowledge, adequate training, and prior experience on the implementation of internet interventions were more prone to present favorable attitudes toward these interventions.

Additionally, participants having more positive attitudes toward internet interventions had a higher probability of recommending internet interventions and online resources to improve the health status of their clients and considering using such interventions in the future. Opposingly, participants presenting more negative attitudes toward internet interventions were less likely to recommend or contemplate using such interventions in the future. No demographic or background factors were significantly associated with attitudes toward internet interventions in this study.

**Table 5:** Factors associated with attitudes toward internet interventions.

Characteristic		Attitudes							Chi-square tests					
		≤36.02		36.03-46.20	46.21-53.48		≥53.49		*P* value		Chi-square		Cramér V	
**Self-reported knowledge^a^ (n=972)**										**<.001**		**χ^2^_12_=109.74**		**.19**
	Completely disagree		63 (4.6)	45 (0.9)		32 (–1.5)		20 (–4.0)						
	Moderately disagree		68 (0.8)	83 (3.3)		60 (–0.4)		40 (–3.8)						
	Neither agree nor disagree		56 (1.0)	50 (–0.1)		56 (1.1)		39 (–2.0)						
	Moderately agree		49 (–3.8)	57 (–2.6)		81 (1.5)		102 (4.9)						
	Completely agree		8 (–2.8)	10 (–2.2)		12 (–1.6)		41 (6.6)						
**Self-reported training^b^ (n=972)**									**<.001**		**χ^2^_12_=64.70**		**.15**	
	Completely disagree		109 (3.1)	103 (2.2)		86 (–0.2)		55 (–5.1)						
	Moderately disagree		64 (–0.5)	64 (–0.5)		77 (1.8)		61 (–0.9)						
	Neither agree nor disagree		41 (–0.1)	43 (0.2)		41 (0)		41 (–0.1)						
	Moderately agree		22 (–2.6)	25 (–2.0)		27 (–1.4)		62 (6.0)						
	Completely agree		8 (–1.6)	10 (–0.9)		10 (–0.9)		23 (3.4)						
**Recommendation^c^ (n=972)**									**<.001**		**χ^2^_3_=42.12**		**.21**	
	No		223 (4.8)	204 (1.1)		194 (–0.2)		166 (–5.7)						
	Yes		21 (–4.8)	41 (–1.1)		47 (0.2)		76 (5.7)						
**Future use^d^ (n=685)**									**<.001**		**χ^2^_3_=123.14**		**.42**	
	No		132 (10.5)	65 (–0.1)		44 (–4.2)		20 (–6.1)						
	Yes		58 (–10.5)	120 (1.0)		133 (4.2)		113 (6.1)						

^a^Adjusted standardized residual frequencies appear in parentheses after observed group frequencies. Original wording: I am familiar with the concept of providing psychological support via the internet. Rated on a 5-point scale: 1=completely disagree to 5=completely agree.

^b^Original wording: I believe to have the necessary training to provide psychological support via the internet. Rated on a 5-point scale: 1=completely disagree to 5=completely agree.

^c^Original wording: Have you ever recommended the use of internet-based psychological support or other online resources in order to improve a client’s health status?

^d^Original wording: Do you expect to use the internet or the telephone to provide psychological support in the future?

## Discussion

### Principal Findings

The aim of this study was to explore Portuguese psychologist knowledge, training, use, and attitudes toward internet interventions, investigate perceived advantages and limitations of such interventions, identify potential drivers and barriers impacting implementation, and study potential factors associated to use and attitudes toward internet interventions.

Results showed that most psychologists were not familiar with internet interventions and had no prior experience using digital technology in the provision of psychological support. Only a minority reported having the necessary training to work in the field. Nevertheless, more than half of nonusers contemplated using it in the future, mainly as blended and pharmacotherapy complementary interventions rather than stand-alone internet interventions. From those who had prior experience implementing such programs, the majority were clinical and health psychologists who used telephone, email, and SMS services as a complement to face-to-face interventions with the purpose of increasing access to information and psychological care when treating mental health disorders such as anxiety or depression. Guided and unguided internet interventions were rarely used in this context. These results are in line with previous studies [15,34,46] that showed a higher acceptance of blended interventions when compared with stand-alone internet interventions but contrast with the reality of countries such as Australia [26], the United Kingdom, and Sweden [34], where the use of internet interventions is widely disseminated. As conceptualized by Topooco et al [34], Portugal, in this domain, may be included in the learners category, since the current experience and practice of e-mental health in the country is very limited.

Although accessibility, convenience, and cost effectiveness are considered important advantages of internet interventions by Portuguese psychologists, their attitudes toward such interventions tend to range from slightly negative to neutral, and a guarded stance is adopted when analyzing the topic. Similar findings were reported by Neves et al [24] in a study assessing the impact of evidence-based practice on the attitudes of Portuguese psychologists toward internet interventions. Perceived barriers and limitations associated to internet interventions implementation may contribute to this predisposition and partly explain the low uptake of these interventions in Portugal.

According to participants in this study, the main barriers to overcome in the implementation of internet interventions were related to limitations on the conceptual comprehension and implementation of self-help techniques by clients, insufficient scientific evidence on the efficacy and cost effectiveness of internet interventions, and difficulties in the adaptation of treatment protocols to the digital format. Although these may be in fact challenges to overcome in some domains, the high number of publications attesting to the efficacy and cost effectiveness of internet interventions based on established treatment protocols and promoting the use of self-help techniques by clients [47-49] refutes these misconceptions. Other important obstacles identified by psychologists participating in this study pertained to patient ICT illiteracy and low adherence. Considering that in 2018 [50], 79% of Portuguese households had access to the internet, 75% of residents in the country aged 16 to 74 years reported using the internet in the previous year, mainly via smartphones, and 67% and 80% used apps and authentication procedures, respectively, ICT illiteracy may still be a barrier in some users over age 55 years and in extremely remote regions, but the necessary conditions to implement internet interventions successfully in Portugal are already in place.

As in previous studies [15,21,27-29], security, confidentiality, and ethical concerns were other important obstacles identified by participants in this research. On one hand, the fact that in 2018 alone several social media companies such as Facebook and Google reported data breaches [51] compromising the personal information of millions of users around the world may contribute to an atmosphere of insecurity and suspicion toward information systems security. On the other hand, the fact that until May 2019, no guidelines for the practice of e-mental health had been published by OPP [52] providing practical and deontological orientation in this domain may have contributed to psychologist reluctance in using information and communication systems in their practice. As we have stated, the provision of psychological services via digital technology has idiosyncrasies and must comply with ethical and security requirements similar to those used in online banking, which may at the same time promote confidence and inhibit the use of internet interventions, depending on psychologist ICT literacy.

Another important aspect relating to the ethics and process of delivering psychological support via the internet that occasionally emerged in this research as potentially affecting implementation pertains to the deleterious effect internet interventions may have on psychological assessment, therapeutic alliance, and crisis management. Like in previous publications [19,28,53], we found that a significant proportion of Portuguese psychologists perceive remote psychological assessment processes as inaccurate, increasing the possibility of misdiagnosis, and unsuitable for crisis management. As reported by Vigerland et al [22] and Perle et al [21], internet intervention adoption by psychologists may depend on disorder type and tend to be rejected for more serious conditions, despite the growing evidence attesting its efficacy in severe disorders [54-56]. Moreover, a disbelief in the possibility of establishing an adequate therapeutic alliance via the internet was reported by approximately one-third of respondents in this study, corroborating the findings of Sucala et al [53]. In that study, clinicians reported less confidence in their skills to develop alliance in e-therapy than in face-to-face therapy, mainly due to anticipated difficulties in reading patient emotions and conveying warmth and empathy in this environment. Although research on this topic is scarce, recent studies suggest therapeutic alliance in internet-based cognitive behavioral therapy is high [57,58] and has the potential of enhancing engagement and rapport in face-to-face psychotherapy when combined [59].

Last, negative attitudes presented both by patients and psychologists toward internet interventions were other important obstacles identified by participants in this research. The assessment of their attitudes exhibited in the context of this study confirmed this assertion. However, although only a few studies focused on the acceptability of internet interventions, the existing literature seems to point in the opposite direction [39,60] and suggests individuals with depression hold more positive attitudes toward such interventions than do psychotherapists [23]. In Portugal, a study focusing on Portuguese women’s acceptance of e-mental health tools during the perinatal period reported good acceptance of internet interventions by this group. Nevertheless, more research focusing on different patient groups is necessary to adequately characterize the attitudes of patients with mental health disorders.

Regarding potential factors associated with Portuguese psychologist use of internet interventions, a significant association was found between previous experience of use and age, years of professional experience, work context, theoretical orientation, attitudes, knowledge, training, and recommendation of internet interventions. Unexpectedly, digital native psychologists (aged 30 years and younger) and psychologists with less than 5 years of professional experience were less likely to have used internet interventions in the past when compared with their middle-aged (aged 41 to 60 years) and more experienced colleagues (16 or more years), a finding that is not justified by a delay entering the labor market, since most of our sample was active and no significant differences were found regarding work status between the different age groups. Furthermore, considering that no significant associations were found between age and attitudes toward internet interventions, this finding might be justified by seasoned psychologists feeling more in control of the therapeutic process and therefore more lenient toward setting rules and more willing to use innovative tools in their practice. Work context also seems to impact internet intervention adoption. Psychologists working at the NHS and in private practices were more likely to include digital technology in the therapeutic process than psychologists working at public services, education/research institutions, and charities. The shortage of mental health professionals [61,62] working at a universal, general, and tendentiously free NHS as is the case in Portugal may possibly burden the class and incentivize the use of creative solutions for patients’ problems. As identified in the research by Venkatesh et al [33], performance expectancy (usefulness, effectiveness, enhancement of quality, diversity of care, and increase in productivity) and facilitating conditions seem to emerge as possible predictors of use. In private practice, the nature of the psychotherapeutic relationship and possible requests from long-term patients living in a globalized digital world probably impose such solutions. This aspect may also justify another surprising finding in this study. Contrasting with previous research [22-24], theoretical orientation was not significantly associated with attitudes toward internet interventions, but dynamically oriented therapists were more likely to have used internet interventions in the past than their colleagues with other theoretical stances. Besides the fact that psychodynamic interventions are typically longer and therefore possibly more challenged by the necessity of including alternative ways of communications in the process, the nonprescriptive nature of psychodynamic psychotherapy may turn dynamically oriented therapists less susceptible to fear of replacement and consequently more open to include digital communication tools in their practice. Although this study did not pursue this line of inquiry, an analysis of participant attitudes toward internet interventions revealed a significant association between previous use and psychologist predisposition toward such interventions, supporting the thesis that attitudes impact adoption. In this study, psychologists presenting more negative attitudes toward internet interventions were less likely to have prior experience using internet interventions when compared with psychologists holding more positive attitudes. Significant associations were also identified between previous use and attitudes toward internet interventions and self-reported knowledge, self-reported training, recommendation, and future use. Psychologists reporting ignorance on the subject and having no training in internet interventions were more likely to present more negative attitudes toward these interventions and have less experience in their implementation. Conversely, psychologists reporting moderate to high knowledge, adequate training, and prior experience in the implementation of these interventions were more prone to present favorable attitudes toward internet interventions, which confirms the findings of Whitfield and Williams [46] and Glueckauf et al [27] and identifies lack of knowledge and training as major barriers to overcome in this context. Naturally, psychologists with previous implementation experience and more positive attitudes toward internet interventions had a higher probability of recommending internet interventions to their clients and contemplating their use in the future.

Considering the prevalence of lifetime mental health disorders in Portugal is above 30% [17], the Portuguese mental health system is failing to comply with World Health Organization recommendations of providing better access and more integrated mental health care to the Portuguese population [63], and Portuguese psychologist attitudes toward evidence-based internet interventions are hindering implementation, mainly due to lack of knowledge and training, immediate corrective actions must be taken. Awareness and training initiatives should be promoted by psychologist associations and universities as an effective means of educating the class, changing professional and student perceptions [25,64,65], and increasing the implementation of ubiquitous strategies such as internet interventions to equitably respond to the mental health care system challenges and limitations. Additional research focusing on the Portuguese e-mental health ecosystem and addressing most prevalent mental health disorders in the country should inform and result from this process.

### Limitations and Future Work

Several limitations must be considered when interpreting our findings. Despite ATIIS good psychometric properties, the fact that the two selected factors—positive and negative attitudes—only account for 44% of the variance explained suggests further research is necessary to understand what other factors might be attributable to psychologist attitudes toward internet interventions. Second, ATIIS online dissemination and the study sample self-selection might have introduced selection bias, limiting the generalizability of the obtained results. ICT illiterate psychologists as well as those presenting more negative attitudes toward internet interventions might not have participated in this study, lowering the response rate and biasing its results. Nevertheless, the study sample may be considered very large, and its demographic and background characteristics are similar to those published on the last census performed by OPP [44], indicating that participants in this study are probably representative of the class. Moreover, findings in our research are concordant with those reported by Neves et al [24], supporting external validity. Third, the exploratory cross-sectional design adopted in this investigation and the fact that no theoretical framework was used to present a structured representation of factors influencing adoption may also be considered a limitation. Additional research adopting structured frameworks and resourcing to mixed-methods research should be performed in order to deeply explore adoption and attitude predictors. To this end, a complementary study, adopting a qualitative descriptive approach consisting of in-depth semistructured interviews with Portuguese psychologists, is being conducted by the research team. Finally, the fact that this study targeted only psychologists and not other stakeholders in the e-mental health ecosystem such as patients/service-users, other health care providers, government bodies, funding/insurance bodies, technical developers, and researchers fails to present a comprehensive picture of the current status and acceptability of e-mental health in Portugal. To address this limitation, the research team is conducting a mixed-methods research study to explore the attitudes of Portuguese breast cancer patients toward internet interventions. In that study, ATIIS was adapted to the target population, and its factor structure will be examined. Future research should characterize the attitudes of other e-mental health ecosystem stakeholders.

### Conclusions

This study investigated the use and attitudes of Portuguese psychologists toward internet interventions and provided insight on the principal barriers hindering implementation in the country. Most Portuguese psychologists were not familiar with and had no training or prior experience using internet interventions. A slightly negative/neutral attitude toward internet interventions was captured, indicating that Portuguese psychologists are cautious toward these interventions and show greater acceptability toward blended treatment interventions compared with stand-alone internet interventions. Lack of knowledge and training are likely the main barriers to overcome for successful implementation and underline the need for awareness and training initiatives focusing not only on internet intervention efficacy and cost effectiveness but also on the practical, relational, technological, ethical, and regulatory requirements this treatment modality entails.

## Appendix

Multimedia Appendix 1 Attitudes Toward Internet Interventions Survey factor analysis (rotated component matrix).

## Abbreviations

ATIIS: Attitudes Toward Internet Interventions Survey
EFA: exploratory factor analysis
EPI: electronic psychological interventions
ICT: information and communication technologies
iNNOVBC: A Guided Internet-Delivered Individually Tailored ACT-Influenced Cognitive Behavioral Intervention to Improve Psychosocial Outcomes in Breast Cancer Survivors
KMO: Kaiser-Meyer-Olkin test
NHS: National Health Service
OPP: Portuguese Psychologists Association (Ordem dos Psicólogos Portugueses)
SMS: short message service
