# Supplementary material for: Portuguese Psychologists' Attitudes Toward Internet Interventions: Exploratory Cross-Sectional Study
Source: JMIR Ment Health. 2020 Apr 6;7(4):e16817. doi: 10.2196/16817 (PMC7171568; doi:10.2196/16817)
Supplement: Multimedia Appendix 1 [file mental_v7i4e16817_app1.docx]

| **ATIIS Factor analysis (rotated component matrix)** | | |
| --- | --- | --- |
| Scale: 1=Completely disagree, to 5=Completely agree. | | |
|  | **Factor Loading** | |
| **Item** | **1** | **2** |
| **Factor 1: Negative attitudes (α =.815)** | | |
| 16. I believe it is easier to learn self-help management strategies with the support of a specialist in person, than via the internet. | .708 | -.129 |
| 12. Internet interventions lead to a loss of control over the therapeutic process, by the mental health specialist. | .674 | -.185 |
| 15. I do not believe it is possible to establish a therapeutic alliance via the internet. | .667 | -.313 |
| 14. I believe that psychological support provided in person by an expert is more efficacious than an Internet Intervention. | .664 | -.165 |
| 7. I believe internet interventions have more disadvantages than advantages. | .647 | -.337 |
| 10. I do not feel comfortable receiving or transmitting sensitive information via the internet, even through a website fulfilling the maximum security requirements (e.g. such as those used in online baking). | .604 | -.233 |
| 6. It is difficult for me to perform psychological assessment at a distance. Consequently, I consider internet interventions increase the risk of misdiagnosis. | .589 | -.036 |
| 18. In a crisis situation, I wouldn’t refer a client to an Internet Intervention. | .459 | -.031 |
| **Factor 2: Positive attitudes (α =.876)** | | |
| 2. The internet is a safe way to provide psychological support. | -.569 | .456 |
| 9. The fact that I can provide psychological support remotely, via the internet, makes this therapeutic modality appealing to me. | -.534 | .462 |
| 3. The internet is an appropriate mean to monitor my patients’ therapeutic progress. | -.505 | .467 |
| 20. If available at my institution, I would consider referring/recommending internet interventions as a complement to pharmacotherapy. | -.138 | .712 |
| 19. If available at my institution, I would consider referring/recommending internet interventions as a complement to face-to-face psychotherapy. | -.190 | .709 |
| 17. The fact that internet interventions allow clients to access informational content, suggestions given by their therapist and self-management strategies instructions, anytime and anywhere, makes this therapeutic modality appealing to me. | -.298 | .637 |
| 4. Internet interventions allow clients to have greater control and influence over their treatment. | -.266 | .632 |
| 11. Some clients express their feelings more easily over the internet than in the presence of a therapist. | -.009 | .614 |
| 13. Some clients feel more comfortable in receiving psychological support via the internet than in person. | -.100 | .606 |
| 5. Internet interventions allow me to perform my job more efficiently. | -.396 | .592 |
| 1. I believe internet interventions are efficacious. | -.428 | .541 |
| 8. Internet interventions are more discrete and confidential than face-to-face psychotherapy. | -.091 | .486 |
| 21. If available at my institution, I would consider referring/recommending internet interventions as a standalone treatment. | -.327 | .375 |
